# The Need for Endowed Research

**Published:** 1905-12-23

**Authors:** 


### The Hospital.

A Journal of

The Medical Sciences and Hospital Administration.

Vol. XXXIX.—No. 1,004. SATURDAY, DECEMBER 23, 1905.

### The Need for Endowed Research.

In the same column of *The Times* of December 18 there appeared two letters whose conjunction is suggestive, and prompts a few words of comment. The first letter is an appeal for funds in support of a Cumberland sanatorium for consumptives. The writer, a president of a society for the prevention of consumption, bases his claim on these facts, among others, that the sanatorium is always full, that several patients have been cured and have returned to their everyday life and work, and that all have been "educated in the conduct of their lives, so as to maintain health and to be no longer a source of infection after leaving the sanatorium." To have achieved this is, of course, to have achieved much, and the philanthropist might well say to himself that in supporting sanatoria he is being wise as well as charitable, which is more than many well-meaning citizens can justly say of themselves. But in the other letter Dr. Sydney Long puts a very pertinent and disturbing question: he asks whether the large sums already subscribed for the open-air treatment of tuberculosis are really being laid out to the best ultimate advantage of the community. "I venture to say," he observes, "that if only a tithe of the money already subscribed had been expended towards a more scientific investigation of the disease, it is more than likely that by this time a remedy would have been discovered." And again: "A cure for consumption has yet to be discovered and it will come from the laboratory; for the credit of English science let every assistance be given to those of our countrymen who are willing and capable of carrying out the necessary investigations to unearth it from the secrets of nature, but who from lack of pecuniary means are now unable to do so."

Here are two antithetical propositions for attacking the problem set by tuberculosis. The sanatorium method caters frankly for the present and the individual: such and such a one is a victim and we must help him in the best way we can. The other method is practically a speculative investment; no one can prophecy with any degree of certainty the discovery of such a specific cure as Dr. Long's optimism leads him to expect. It is no wonder that untaught human nature instinctively inclines towards the individual and the present, and prefers to apply its charity to the amelioration of visible suffering rather than to the problematical emancipation of posterity. None the less it is the manifest duty of the initiated, the scientifically provident, to keep constantly before the public eye the larger horizon to which Dr. Long invokes attention.

If there is no doubt that the open-air treatment of consumption makes largely for the improvement of sufferers from this malady and in a less degree for their cure, it is equally certain that advantage upon such lines is within the reach of none but a lamentably small proportion of the total army of martyrs. The economic conditions which govern sanatorium treatment, mitigated though they be by the extraordinary beneficence of the public in this country, nevertheless remain, and must remain, a drag upon the extended use of sanatoria by the poor. It is natural, therefore, that scientific anticipation should reach out for some more active and more specific armament against the ravages of our old enemy; some method of treatment which, like Behring's diphtheria antitoxin, may rapidly reduce to relative insignificance the horrors of the plague it is intended to antagonise.

Unhappily, the problem set by septicæmic diseases like tuberculosis—diseases, that is to say, marked by a wide dissemination of the infecting organism by the blood-stream, seems to be far harder of solution than that offered by such a disease as diphtheria, in which the infecting organism never enters the circulation. In the latter case all that is required is a chemical antidote for the diffusible poisons generated in the local lesion, but in the case of tuberculosis the need is of an anti-microbic serum which will attack the bacillus in its lair; and such sera remain too much in the sphere of experimental triumphs. At the same time the tendency of research is in the direction of specificity in the cures for microbic infections, and the importance of nursing and cultivating research along these lines should need little emphasis. But it does need emphasis, for the public remains very ignorant of the rare elements whose combination is necessary to success in the department of bacteriological experiment. Such investigation demands in the investigator, firstly, originality, which is almost priceless in most markets of the world; it demands a degree of scientific knowledge and precision which can only be the fruit of a long, laborious and expensive apprenticeship; and it demands an undivided and continuous attention. It is easily seen how far we are at present from possessing investigators thus endowed; men of originality we have, a few of them men fitted in every intellectual respect to achieve untold successes in the field of such research. Yet all too often they are men whose activities are hobbled by a vulgar but urgent need of pence; men whose shallow purses compel them to traffic in elementary teaching at our medical schools. Nasmyth hammers used for breaking walnuts.

One might have said that the problem of tuberculosis was momentous enough to command the attention of the State; but our statesmen care for none of these things, though the initiative of King Edward may stir them to activity one of these days. In the meanwhile there remains a magnificent opportunity for some wise man to endow and equip a laboratory of research in such a fashion as to attract the high intellectual endowment which is a prime essential in the field of experimental therapeutics.

